# Types of apathetic depressions formed in endogenous diseases.

**DOI:** 10.1192/j.eurpsy.2023.2120

**Published:** 2023-07-19

**Authors:** A. Barkhatova, S. Sorokin

**Affiliations:** Department of endogenous mental disorders and affective states, Mental health research center, Moscow, Russian Federation

## Abstract

**Introduction:**

The topic of apathetic depression remains insufficiently studied to date. This is due to the lack of unity of views on the definition of apathy and discrepancies in the definition of it’s phenomenological boundaries.

**Objectives:**

The study of the psychopathological features of endogenous apathetic depression, the development of a typology of apathetic depression based on differences in the structure of apathetic phenomena.

**Methods:**

The study included 70 patients (31 male, 39 female) suffering from endogenous apathetic depression in the framework of affective diseases – recurrent depressive and bipolar affective disorder (25 cases) and schizophrenia (45 cases). Clinical-psychopathological and clinical-catamnestic methods were used.

**Results:**

Apathy in depressions differs in the disproportion of the representation of individual components of the apathetic syndrome. Based on the revealed differences, typological varieties of endogenous apathetic depressions were identified: with a predominance of a decrease in interests (30 cases, 42.9%); with a predominance of a decreased initiative (13 cases, 18.6%); with a predominance of a motivational decrease (27 cases, 38.6%). Last group, based on the degree of complicity of the adynamic component, was divided into apatheticadynamic and simple apathetic subtypes. Depression dominated by a decrease in interests was characterized by the predominance of the emotional component in the structure of apathy, consumatory anhedonia, but without loss of the ability to be involved in activities and volitional impairment. Depressions with a predominance of decreased initiative are characterized by the leading role of the cognitive component of apathy with the inability of patients to occupy themselves independently, with minimal severity of anhedonia and the formation of moral hypochondria phenomena. Depressions with a predominance of motivational decline are characterized by the dominance of the behavioral component of apathy with the loss of stimuli and motives for activity, anticipatory anhedonia, volitional deficit, with the inability to engage in any activities.

**Conclusions:**

Apathetic disorders formed within the framework of endogenous depressions are characterized by heterogeneity, which is associated with an uneven representation of declining interests, the ability to show initiative and the motivational component of apathy.

**Disclosure of Interest:**

None Declared

